# TLR Agonists as Mediators of Trained Immunity: Mechanistic Insight and Immunotherapeutic Potential to Combat Infection

**DOI:** 10.3389/fimmu.2020.622614

**Published:** 2021-02-18

**Authors:** Allison M. Owen, Jessica B. Fults, Naeem K. Patil, Antonio Hernandez, Julia K. Bohannon

**Affiliations:** ^1^ Department of Anesthesiology, Vanderbilt University Medical Center, Nashville, TN, United States; ^2^ University of Texas Southwestern Medical School, Dallas, TX, United States; ^3^ Department of Pathology, Microbiology and Immunology, Vanderbilt University Medical Center, Nashville, TN, United States

**Keywords:** trained immunity, immunomodulators, Toll-like receptors (TLRs), TLR agonists, nosocomial infections, immunosuppression, antibiotic resistance, vaccine adjuvant

## Abstract

Despite advances in critical care medicine, infection remains a significant problem that continues to be complicated with the challenge of antibiotic resistance. Immunocompromised patients are highly susceptible to development of severe infection which often progresses to the life-threatening condition of sepsis. Thus, immunotherapies aimed at boosting host immune defenses are highly attractive strategies to ward off infection and protect patients. Recently there has been mounting evidence that activation of the innate immune system can confer long-term functional reprogramming whereby innate leukocytes mount more robust responses upon secondary exposure to a pathogen for more efficient clearance and host protection, termed trained immunity. Toll-like receptor (TLR) agonists are a class of agents which have been shown to trigger the phenomenon of trained immunity through metabolic reprogramming and epigenetic modifications which drive profound augmentation of antimicrobial functions. Immunomodulatory TLR agonists are also highly beneficial as vaccine adjuvants. This review provides an overview on TLR signaling and our current understanding of TLR agonists which show promise as immunotherapeutic agents for combating infection. A brief discussion on our current understanding of underlying mechanisms is also provided. Although an evolving field, TLR agonists hold strong therapeutic potential as immunomodulators and merit further investigation for clinical translation.

## Introduction

Nosocomial infections, or healthcare associated infections (HCAI), represent a significant cause of global morbidity and mortality, and the United States is no exception. Each year, approximately one out of 25 hospitalized patients in the United States is diagnosed with at least one infection related to hospital care (1). Infection leading to sepsis remains one of the leading causes of death in U.S. hospitals, affecting more than 1.7 million and causing 270,000 deaths annually. Sepsis is also a major contributor to re-hospitalizations and is one of the most expensive conditions treated in U.S. hospitals, costing more than $2 billion per year (2–4). The overall 30-day mortality rate for patients in the intensive care unit (ICU) is approximately 20%, and for patients with sepsis and accompanying organ dysfunction this statistic is 30%–50% (5). Despite advancements in healthcare overall, this clinical outcome has not improved over the past 25 years (6).

Critically ill patients are at a significantly increased risk of infection due to injury- or illness-induced immune dysfunction and pathogen exposure through invasive life-saving procedures in the healthcare setting (7). Further complicating the risk of HCAI is the continuing rise of antibiotic-resistant pathogens. Infections secondary to resistant pathogens are one of the most critical threats to modern medicine, and this situation is being exacerbated by dwindling effective treatment options (8). The United States has more than 2.8 million antibiotic-resistant infections annually, resulting in more than 35,000 deaths (1). Solely focusing on the development of new antibiotics is not a permanent solution as pathogens will continue to evolve and become resistant to new drugs (9). Thus, immunomodulatory therapies that boost host immune responses and protect immunocompromised patients against infections are a highly attractive strategy.

One promising approach to restore immune responses relies on the induction of innate immune memory, also termed trained immunity. Classically, the role of the innate immune system is to recognize a pathogen and mount a broad and rapid response with immunological memory being considered specific to the adaptive immune system. Recent evidence demonstrates that innate immune cells also display long-term adaptive characteristics after initial challenge with pathogens or their products, which results in enhanced capacity to eliminate infections upon subsequent challenge (10, 11). Trained immunity refers to the phenomenon of activating the innate immune system through exposure to pathogen associated molecular patterns (PAMPs), triggering long-term functional reprogramming by which innate leukocytes mount an enhanced antimicrobial response upon exposure to a secondary microbial pathogen (12). This protection is broad whereby the host is resistant to an array of pathogens for weeks to months once the altered functional state of innate immune training is initiated. Important to note, the terminology referring to different adaptive programs of innate immunity has evolved with the field. Although used somewhat interchangeably in the literature, a consensus has recently been made to clearly differentiate between the four different adaptive programs: differentiation, priming, tolerance, and training (13). Trained immunity specifically refers to the phenomenon in which the activation status of innate cells returns to baseline after primary stimulation prior to the secondary stimulation. Nevertheless, this active new field of research is rapidly evolving, an arm of which is aimed at taking advantage of trained immunity as an innovative strategy to combat infection (14).

Emerging evidence suggests that Toll-like receptor (TLR) agonists are a promising class of immunomodulatory agents that confer long-term protection against subsequent infectious challenge *via* enhanced innate immunity (15, 16). TLRs play a crucial role in activation of innate immune responses by recognizing PAMPs which then trigger downstream signaling pathways and ultimately stimulate the production of proinflammatory cytokines and type I interferons. Several TLR agonists are recognized widely for their vaccine adjuvant properties and several are FDA-approved for this use, but their ability to induce trained immunity is becoming more recognized. Here, we review recent progress in our understanding of mechanisms of TLR agonist-mediated trained immunity and its strong potential for clinical translation to protect patients against life-threatening infection.

## Toll-Like Receptor Signaling Pathways

As the first line of defense against pathogens, the innate immune system utilizes pattern recognition receptors (PRRs) to rapidly detect microbes and deploy antimicrobial responses. TLRs are a well characterized family of PRRs comprised of 10 members in humans (TLR1-TLR10) and 12 members in mice (TLR1-9, TLR11-13) that are expressed in innate immune cells (i.e. dendritic cells, DCs; and macrophages) and non-immune cells (*i.e.* fibroblasts and epithelial cells) (17). These receptors are synthesized in the endoplasmic reticulum (ER), processed in the Golgi apparatus, and transported to the plasma membrane or intracellular compartment depending on the localization of the PAMP they recognize (18, 19). TLRs detect a wide array of PAMPs, including Gram negative and positive bacteria, viruses, flagellin proteins, lipids, nucleic acids, and damage-associated molecular patterns (DAMPs). This is in part accomplished by receptor localization to the cell surface or intracellular compartments (20). TLRs which recognize nucleic acids are localized to intracellular compartments for decreased risk of contact with “self” nucleic acids whereas cell surface TLRs largely recognize microbial membrane compartments and therefore do not require this protective strategy (21).

**Figure 1:**
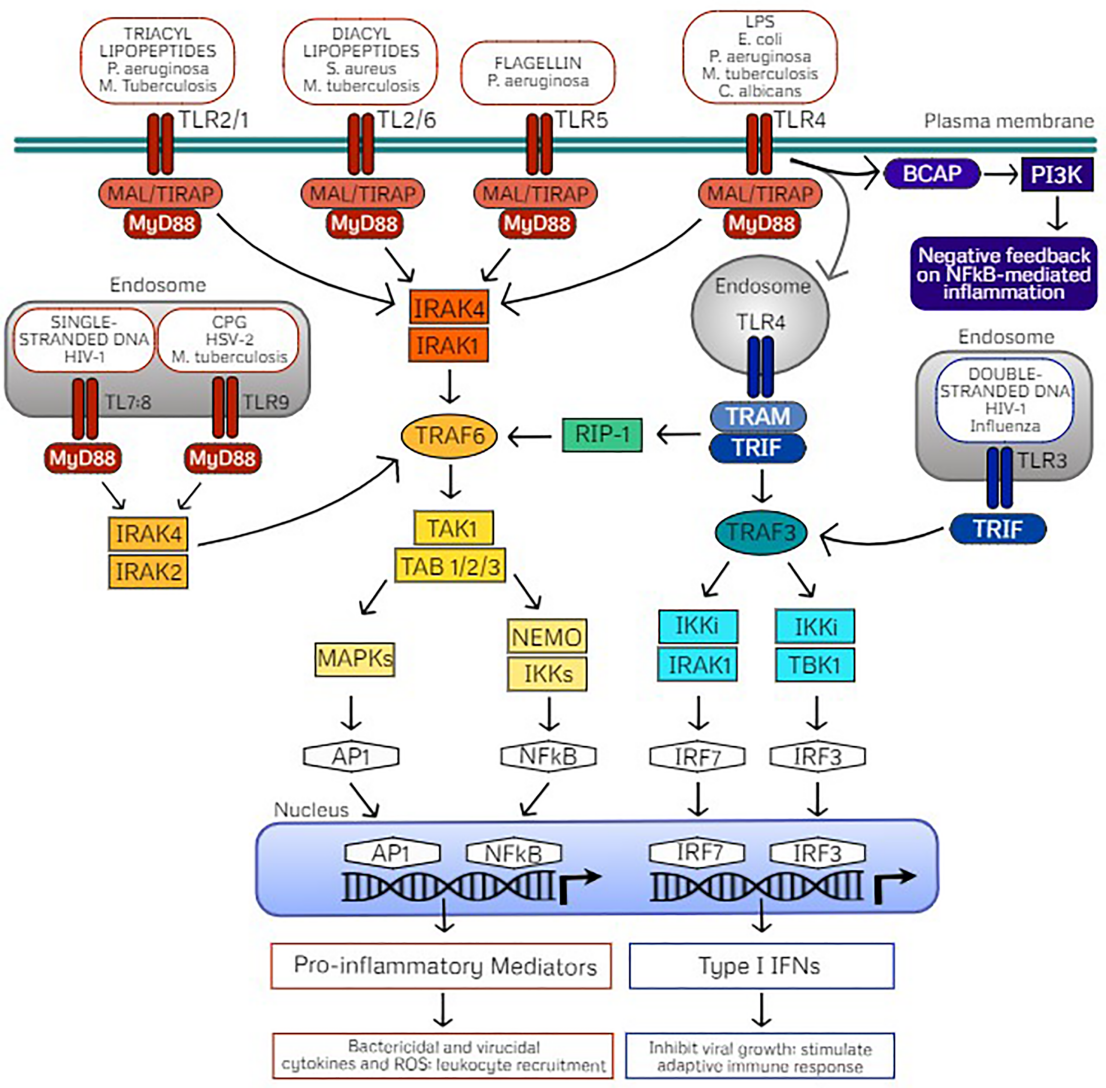
TLR signaling pathways mediated through MyD88- or TRIF-dependent cascades. Cell surface Toll-like receptors (TLRs) include TLR4 and TLR5 which form homodimers upon recognition of their classic ligands lipopolysaccharide (LPS) and flagellin, respectively. TLR2 is also localized on the cell surface which heterodimerizes with TLR1 or TLR6, dependent on ligand recognition with either triacyl or diacyl lipopeptides. TLR recognition of PAMPs on common pathogens are indicated. These cell surface TLRs signal *via* the adaptor protein myeloid differentiation primary response protein (MyD88) through MyD88-adapter-like protein (MAL) also referred to as TIR Domain Containing Adaptor Protein (TIRAP). Intracellular TLRs include TLR9, stimulated by agonist CpG oligodeoxynucleotides (CPG) and the heterodimer TLR7/TLR8, stimulated by single stranded DNA (ssDNA) which signal through direct interaction with MyD88. Activation of MyD88 signaling induces phosphorylation of IL-R-associated kinases (IRAKs) dependent on TLR localization which in turn interacts with TNF receptor-associated factor 6 (TRAF6) and downstream activation of the TAK1 (transforming growth factor β–activated kinase 1)/TAB (TAK1-binding protein) complex. The TAK1/TAB complex activates mitogen-activated protein kinases (MAPKs) or the IkB kinase (IKK) complex of which NF-κB essential modulator (NEMO) is the regulatory subunit. These signaling events activate transcription factors activator protein-1 (AP1) and nuclear factor-κB (NFκB), which translocate into the nucleus for transcription of inflammatory mediators. As a result, pro-inflammatory cytokines and reactive oxygen species drive bacterial killing and limit viral replication as well as stimulate leukocyte recruitment to clear the infectious pathogen. Alternatively, TLR3 recognizes double stranded DNA (dsDNA) and uniquely signals through the adaptor protein Toll/IL-1R (TIR) domain-containing adapter producing interferon-β (TRIF) which interacts with TRAF3. TLR4 also activates TRIF through endocytosis, which activates TRIF signaling *via* the sorting adaptor protein TRIF-related adaptor molecule (TRAM). Activated TRAF3 signals through IKKi/IRAK1 or IKKi/TBK1 which activate the transcription factors interferon-regulatory factor (IRF) 7 and IRF3, respectively, which translocate to the nucleus for transcription of type I interferons (IFNs). Type I interferons act to inhibit viral replication as well as stimulate adaptive immunity. TRIF-dependent TLR4 signaling can also activate TRAF6 *via* receptor interacting protein (RIP)-1 for late NFκB signaling. B-cell adaptor for phosphatidylinositol 3-kinase (PI3K) (BCAP) also seems to be an adaptor protein that confers negative feedback on NFκB-mediated inflammation *via* PI3K as a regulatory mechanism.

TLRs are composed of a horseshoe-like leucine-rich repeat (LRR) ectodomain which interacts with the respective PAMP or DAMP, a transmembrane helix domain, and a cytoplasmic Toll/IL-1 receptor (TIR) domain which is involved in activation of downstream signaling (22, 23). Upon ligand binding, TLRs homo- or hetero-dimerize which dictates recruitment of specific TIR containing adaptor proteins for activation of downstream signaling. TLRs signal either by recruiting the adapter molecule myeloid differentiation primary response differentiation gene 88 (MyD88) or the MyD88-independent Toll/IL-1R (TIR) domain-containing adapter producing interferon-β (TRIF) signaling. Initiation of MyD88- or TRIF-dependent signaling activates mitogen-activated protein kinases (MAPKs) and IkB kinases (IKKs) that then activate transcription factors to regulate the expression of pro-inflammatory cytokines and chemokines as well as type I interferons (**Figure 1**). Therefore, these pathways trigger unique antimicrobial defenses to confer protection against diverse pathogens. MyD88- and TRIF-dependent signaling cascades are important to consider in the understanding and application of TLR-mediated trained immunity.

### MyD88-Dependent Signaling Cascades

Upon ligand binding, all TLRs except TLR3 initiate downstream signaling by recruiting adaptor protein MyD88 either directly (TLR9, TLR11, TLR13, TLR7/TLR8, and TLR2/TLR10) or indirectly *via* the sorting adaptor TIR domain containing adaptor protein (TIRAP; also termed MyD88-adaptor-like, MAL; TLR4, TLR5, TLR2/TLR1, and TLR2/TLR6) (18, 24). TIRAP binds with different lipids depending on TLR localization which mediates assembly of kinases (IRAK4 and either IRAK2 or IRAK1) termed the “Myddosome” (17, 25, 26). Through formation of this oligomeric signaling complex, the kinase domains of IL-1 receptor-associated kinases (IRAKs) are phosphorylated. Activated IRAK1 associates with TRAF6 and the TAK1 protein kinase complex which culminates in activation of the transcription factors nuclear factor-κB (NF-κB) and activator protein-1 (AP-1) *via* IKKs and MAPK, respectively. Thus, MyD88-dependent TLR signaling results in translocation of NF-κB and AP-1 to the nucleus for production of pro-inflammatory mediators, playing a critical role in triggering an inflammatory response for defense against an invading pathogen.

Beyond its role in stimulating inflammation, MyD88 is a key regulator of phagocytosis of bacteria by macrophages (27) and DCs (28) *via* IRAK4 and p38 MAPK (29) which lead to the expression of scavenger receptors. This pathway also influences phagocytosis *via* NADPH oxidase assembly and thus superoxide production for bacterial killing (30). Upon TLR4 stimulation by lipopolysaccharide (LPS) in macrophages, it has been found that MyD88 signaling also activates Src tyrosine kinase *via* the cytoplasmic protein EGF receptor pathway substrate number 9 [Eps8 (31)] which causes actin cytoskeleton rearrangement. Although alternate MyD88-independent pathways have been identified [actin-Cdc42/Rac pathway (32); CD14/complement receptor 3 (33)], the MyD88 pathway seems to be the main driver of phagocytosis of *Borrelia burgdorferi* (34). It also plays a role in phagocytosis of fungal pathogens, although this differs depending on the fungal challenge (35). The role of MyD88 signaling in innate immunity is highlighted by high incidence of life-threatening infection in patients with MyD88 and IRAK4 deficiencies (36, 37).

### TRIF-Dependent Signaling Cascades

TLR3 exclusively signals through the TRIF-dependent pathway through direct interaction, whereas TLR4 uniquely signals through MyD88 at the cell surface or through TRIF (also referred to as TICAM-1) upon internalization of the receptor complex after ligand binding (20). Trafficking of TLR4 to the endosomal compartment is dependent on CD14 (38, 39). After TLR4 endocytosis, the recruitment of the adaptor protein TRAM (also termed TICAM-2) is coordinated with the release of the TIRAP-MyD88 complex (19, 40). From the endosomal compartment, TLR3 and TLR4 associate with the TRAM-TRIF complex which interact with TNF receptor-associated factor 6 (TRAF6) and lead to activation of NF-κB or AP-1 and downstream production of inflammatory cytokines. Alternatively, interaction with TRAF3 induces interferon regulatory factors 3 (IRF3) or IRF7 and downstream production of type I interferons (17, 40). Briefly, TRAF6 activates the TAK1 complex that subsequently activates NF-κB and MAPKs *via* the IKK family member NF-kB essential modifier (NEMO). On the other hand, TRAF3 recruits TBK1 and IKKi resulting in phosphorylation of IRF3 or IRF7 which dimerize and translocate to the nucleus to induce to transcription of IFNs. Additional intricacies of the TRIF-dependent signaling cascade are reviewed by Ullah et al. (18). Production of type I IFNs is most often associated with defense against double-stranded RNA (dsRNA) viruses; however, they are also important for response to single-stranded RNA (ssRNA) viruses, DNA viruses, and bacteria (41, 42). TRIF signaling seems to play a role in activation of the adaptive immune system *via* T cell stimulation. Importantly, TRIF signaling mediates caspase activation, apoptosis, and necroptosis which may play a role in removing infected cells, thus preventing pathogen dissemination *via* cell death (43–45).

### Balance Among MyD88- and TRIF-Dependent-Signaling

Activation of MyD88-dependent and TRIF-dependent signaling cascades allows for immune functional responses specific to the pathogen sensed by TLR ligand binding, as discussed above. Thus, these pathways are subject to several regulatory strategies for the balanced production of inflammatory cytokines and type I IFNs for elimination of pathogens but also to control the magnitude of the response to prevent pathogenic inflammation and autoimmune disease (46). Such regulatory controls of TLR-mediated inflammatory responses include cooperation with coreceptors, post-translational modifications, cellular trafficking, and negative feedback, which are reviewed in detail by Leifer and Medvedev (47). Commonly, regulatory molecules (1) interfere with signaling complex formation *via* TIR domain-containing molecules, (2) prevent association of TRAF6 or TRAF3 with their respective signaling complexes *via* deubiqutinases (48–50), (3) competitively inhibit downstream signaling (51), or (4) provide mRNA stability of signaling molecules regulated by miRNAs (50), or cytokines by RNA-binding proteins (52).

Interestingly, both MyD88-dependent and TRIF-dependent pathways are required for maximal antimicrobial responses upon LPS-activation of TLR4 (53), demonstrating that they work in concert rather than being redundant. As such, TLR4 signaling is tightly controlled by localization, internalization upon ligand binding (thus acting as a temporal regulator), as well as influence of regulatory molecules. In addition to MAPK and NF-κB pathway activation upon TLR stimulation, the PI3K pathway is also activated *via* B-cell adaptor for PI3K (BCAP) in macrophages (54, 55) and DCs (56). BCAP/PI3K signaling serves as a negative feedback arm which limits NF-κB induced inflammation and acts as an endogenous regulatory mechanism (57). Additionally, the adaptor TRAF3 has been found to play an inhibitory role on TLR-mediated MAPK activity through preventing the release of the TAK1 signaling complex (58, 59), while peroxiredoxin-1 (PRDX1) attenuates NF-κB activation *via* attenuation of ubiquitin-ligase activity of TRAF6 (60). Understanding these endogenous negative feedback mechanisms will be highly useful while translating TLR-mediated trained immunity for clinical application to protect patients against infection with careful attention as to limit inflammatory responses.

## Toll-Like Receptor Agonist-Mediated Trained Immunity and Protection Against Infection

### Innate Immune Cell Types Which Drive Toll-Like Receptor-Induced Trained Immunity

TLR agonists have exhibited highly attractive immunomodulatory properties whereby they induce augmentation of cell recruitment, antimicrobial effector functions (*i.e.* phagocytosis, respiratory burst, production of proinflammatory cytokines and chemokines), bacterial clearance, attenuate inflammation, and trigger cross-protection to infection with clinically relevant pathogens (61). Importantly, trained immunity is not dependent on T and B lymphocytes as evidenced by preserved protection against several models of infection in transgenic RAG2 knockout mice comparative to survival benefit observed in wild type animals (62, 63).

**Figure 2:**
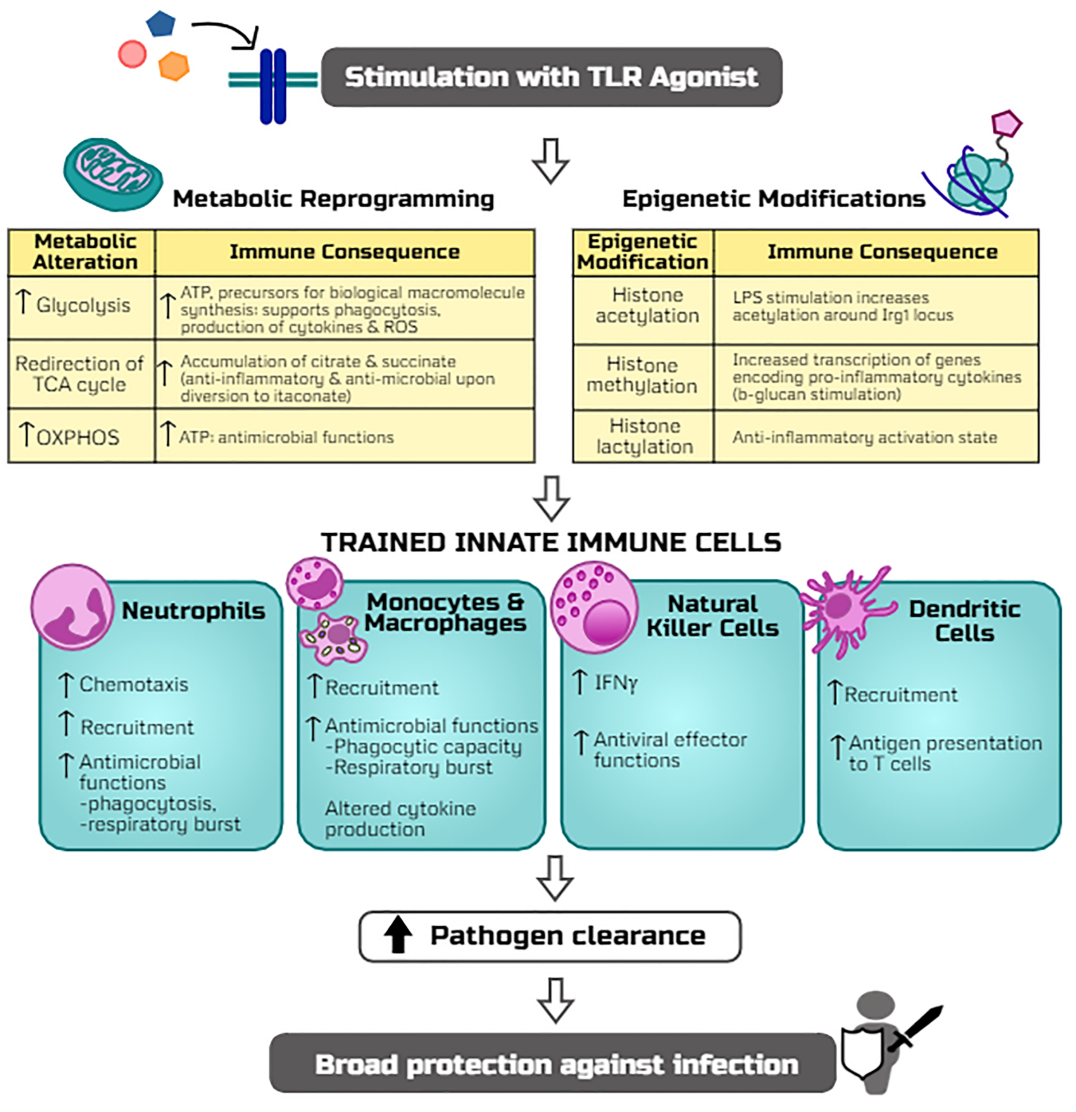
Potential mechanisms by which TLR agonists trigger trained immunity and host resistance to infection. The stimulation of TLR signaling induces metabolic reprogramming (including alterations in glycolysis, TCA cycle, and oxidative phosphorylation, i.e. OXPHOS) and epigenetic modifications (histone acetylation, methylation, and lactylation) which rewire innate leukocytes for more robust antimicrobial functions upon a secondary infectious challenge. Adaptations of innate cell programs thereby allow for more efficient clearance of pathogens and thus protection against a broad array of infections.

To date, TLR-mediated innate immune cellular responses have largely been studied in monocytes, macrophages, and natural killer (NK) cells which show long-term functional reprogramming with increased responses to secondary stimulation by bacterial, parasitic, or viral microbes (12, 62, 64) (**Figure 2**). As the ‘first responders’ to infection, neutrophils play a key role in TLR-mediated resistance to infection *via* increased recruitment and function (65–67). Similarly, activation of macrophages by TLR signaling results in increased antimicrobial effector functions (phagocytic capacity, respiratory burst, altered production of inflammatory mediators) (63). Importantly, both neutrophils and macrophages are required for TLR4-mediated resistance to infection. DCs can be activated by TLR signaling or secondary to TLR-activation of NK cells which ‘bridge the gap’ between innate and adaptive immunity whereby activated and matured DCs migrate to the lymph nodes and subsequently activate naïve T-cells (15, 68). Thus, immunomodulation of DCs by TLR signaling may prove to be an effective vaccine adjuvant strategy (69). Continued investigation regarding innate immune cell responses to TLR-mediated trained immunity will help refine therapeutic strategies to address specific clinical scenarios.

### Immunomodulation *via* Targeting Cell Surface Toll-Like Receptors

Agonists which stimulate cell surface TLRs 2, 4, and 5 have been widely studied for their potential as immunomodulators and conferring host resistance to infection. Such studies are discussed below and are summarized in **Table 1**.

**Table 1:** Agonists which trigger trained immunity *via* cell surface TLRs.

TLR	Agonist	Route of Administration	Infectious Model	Antimicrobial Response	Reference
TLR2	MALP-2	*i.t.*	*S. pneumoniae* (*i.n.*)	↑ Leukocyte recruitment	Reppe et al. (70)
				↓ Bacteremia in lung parenchyma	
		*i.t.* prior to *S. pneumoniae* infection	*Influenza A* (*transnasal*) *+ S. pneumoniae (i.n.)* superinfection	↓ Pulmonary bacterial load	Reppe et al. (71)
		Assay media of BMDMs	*M. tuberculosis* inoculation of BMDMs	↓ Bacterial growth	Palma et al. (72)
				↑ Nitric Oxide	
	Pam_2_Cys	*i.n.*	Influenza *A* (*i.n.*)	↑ Neutrophil and macrophage recruitment	Tan et al. (73)
				↑ Pro-inflammatory cytokines	
TLR4	LPS	*i.p.*	*E. coli* (*i.p.*)*; P. aeruginosa* (*i.p.*)	↑ Bacterial clearance	Landy & Pillemer (74)
		*i.p.*	*P. aeruginosa* (*i.p.*)	↑ Bacterial clearance (systemic and lungs)	Varma et al. (75)
				↓ Pro-inflammatory cytokines (plasma)	
		*i.p.* and *i.v.*	*S. aureus* (*i.v.*)	↑ Bacterial clearance	Murphey et al. (61)
				↓ Pro-inflammatory cytokines (plasma)	
	MPLA	*i.p.*	*P. aeruginosa (*topical inoculation of burn wound or *i.p.*)	↑ Bacterial clearance	Romero et al. (65)
				↑ Leukocyte recruitment	
			Polymicrobial abdominal sepsis (CLP surgical model)	↓ Pro-inflammatory cytokines (plasma)	
		*i.p.*	*P. aeruginosa (topical inoculation of burn wound)*	↑ Neutrophil mobilization & recruitment to site of infection	Bohannon et al. (67)
		*i.p.*	*P. aeruginosa (i.p.)*	↑ Neutrophil & macrophage recruitment	Fensterheim et al. (76)
				↓ Pro-inflammatory cytokines (plasma)	
		*i.v.*	*S. aureus* (*i.v.*)	↑ Bacterial clearance	Fensterheim et al. (63)
			*C. albicans (i.v.)*	↓ Pro-inflammatory cytokines (plasma)	
			* *	↓ Organ injury (kidney)
	PHADs	*i.p.*	*P. aeruginosa (i.p.)*	↑ Bacterial clearance	Hernandez et al. (77)
				↑ Leukocyte recruitment	
		*i.v.*	*S. aureus (i.v.)*	↑ Antimicrobial functions	
				Attenuates systemic and local inflammation	
	AGP	*i.n.*	Influenza *A (i.n.)*	↑ Pathogen clearance	Baldridge et al. (78)
		*i.v.*	*L. monocytogenes* (*i.v.*)		
		*i.n.*	*F. tularensis* (*inhalation*)	↑ Cytokine & inflammatory responses	Lembo et al. (79)
				↑ Bacterial clearance	
		*i.n.*	*Y. pestis* (*i.n.*)	↑ Bacterial clearance (lungs)	Airhart et al. (80)
	fmOMV	*i.n.*	H1N1, PR8, H5N2, H5N1	↑ Type I IFNs	Bae et al. (81)
				↑ Macrophage recruitment	
	FimH	*i.n.*	Influenza A (i.n.)	↑ Neutrophil recruitment	Abdul-Careem et al. (82)
				↓ Organ injury (lung)	
		*Transurethral instillation*	Uropathogenic E. coli (UPEC) or	↑ Bacterial clearance (bladder)	Habibi et al. (83)
			*P. mirabilis*		
TLR5	Flagellin	*i.p.*	Rotavirus (oral inoculation)	↓ Viral load	Zhang et al. (84)
				↓ Viral replication	
		*Sublingual*	*S. pneumoniae* (*i.n.*)	↑ Neutrophil recruitment	Munoz-Wolf et al. (85)

i.n., intranasal administration; i.p., intraperitoneal injection; i.t., intrathecal administration; i.v., intravenous injection.

#### Toll-Like Receptor 2

TLR2 recognizes lipid-containing PAMPs of Gram positive bacteria (*i.e.* lipopeptides, peptidoglycan), as well as viral (*i.e.* HSV glycoproteins) and fungal (*i.e.* zymosan) pathogens, and upon activation it forms heterodimers with TLR1, TLR6, or other cell surface molecules such as Dectin-1 and CD36 (86). Both TLR2/TLR1 and TLR2/TLR6 result in MyD88-dependent signaling *via* MAL/TIRAP; however, the TLR2/TLR1 heterodimer is activated by triacyl lipopeptides whereas the TLR2/TLR6 heterodimer is stimulated by diacyl lipopeptides (87). Several TLR2 agonists have shown promising immunomodulatory effects.

First isolated from *Mycoplasma fermentans* in 1997 (88), macrophage-activating lipopeptide-2 (MALP-2) has become a well-studied immunomodulator which activates TLR2/TLR6 heterodimer. When administered 24 h prior to challenge with *Streptococcus pneumoniae*, MALP-2 treatment reduced bacterial load and enhanced leukocyte migration in the lungs (70). Interestingly, treatment of influenza A virus-infected mice with MALP-2 prior to challenge with *S. pneumoniae* enhanced leukocyte recruitment and reduced bacterial load in the lungs, and was associated with increased survival and improved body condition (71). MALP-2 immunomodulation is presumably driven by its ability to rapidly stimulate neutrophil chemotactic activity followed by induction of monocyte chemoattractant protein-1 (MCP-1) activity in the lungs (89). Further, MALP-2 induces production of proinflammatory cytokines (IL-6, TNF-α) and chemokines (macrophage inflammatory protein-1α and -1β; MIP) (89, 90). Palma et al. also postulated that the microbicidal effect which they observed of MALP-2 on *Mycobacterium tuberculosis* was mediated by nitric oxide (NO) production (72).

As MALP-2 demonstrates attractive immunomodulatory potential, several synthetic analogs of the molecule, termed palmitoylated peptides, have been investigated in the recent decade. The peptide dipalmitoyl-S-glyceryl cysteine (Pam_2_Cys) targets TLR2/TLR1, whereas the peptide tripalmitoyl-S-glyceryl cysteine (Pam_3_Cys) stimulates TLR2/TLR6. These peptides and their derivatives hold strong potential as vaccine adjuvants, with Pam_2_Cys seeming to be more ideal due to increased solubility and potency compared to Pam_3_Cys (91, 92). A body of evidence suggests that immunostimulation with Pam_2_Cys provides immediate protection against acute infection with influenza virus but also allows for the development of specific immune responses for long-term protection (73). Such protection is mediated *via* activation of DCs, increased leukocyte recruitment, and increased production of inflammatory cytokines (73). Mifsud and colleagues demonstrated that a derivative of Pam_2_Cys mediates potent anti-viral activity against influenza infection but also protects against secondary infections with *S. pneumoniae* by reducing bacterial burden and inflammation (93). Although these compounds display promise, their synthesis is expensive; thus, recently a novel inexpensive synthesis strategy of an N-acetylated Pam_2_Cys analog has been developed, which seems to maintain high potency (94).

The potential application of another TLR2/TLR6 agonist, GSK3277329, was studied in the context of chemotherapy-induced neutropenia which demonstrated that repeated daily injections of the compound for 2 weeks effectively restored neutrophil loss in monkeys given chemotherapy treatment (95). This evidence further illustrates the potential application of TLR agonists in the context of protecting immunocompromised patients from potentially life-threatening infection by boosting innate immunity.

#### Toll-Like Receptor 4

TLR4 recognizes lipopolysaccharide (LPS) and uniquely signals through both MyD88- and TRIF-dependent pathways. Discovered in 1956 by Landy and Pillemer, mice treated with LPS were resistant to subsequent challenge with Gram negative pathogens (74). Following this work, it became evident that LPS conferred resistance to a broad array of microbes beyond Gram negative bacteria (75, 96) to include Gram positive *Staphylococcus aureus* (61) and fungal pathogens (97) as well as polymicrobial sepsis (98). LPS-mediated resistance to infection is associated with reduced bacterial burden (75, 99), increased leukocyte recruitment (61, 100), and attenuated inflammation (101).

Despite several studies demonstrating its potential therapeutic benefit, the application of LPS as an immunomodulator for translation to the clinical scenario was largely abandoned due to its toxicity (16, 102). More recently, derivatives of LPS as well as synthetic molecules have demonstrated potent induction of trained immunity with significantly reduced toxicity, thus holding strong therapeutic potential. Notably, monophosphoryl lipid A (MPLA) is structurally identical to LPS with the exception of the cleaved C1 phosphate group from lipid A which reduces its toxicity 100-fold (103, 104). Binding of MPLA by TLR4 induces both MyD88- and TRIF-dependent signaling, although MyD88 signaling seems to be predominant (105). Similarly to protection conferred by LPS immunomodulation, administration of MPLA prior to infectious challenge provides a survival benefit to an array of pathogens including Gram negative *P. aeruginosa*, Gram positive *S. aureus*, viral influenza, and fungal *C. albicans* (63, 65, 106). Importantly, MPLA also protects burn-injured mice from wound infection with the clinically relevant pathogen *P. aeruginosa* (67) and in large animals (sheep (107) demonstrating its capacity to induce protection in the immunocompromised host. MPLA-driven survival benefit lasts for at least 10 days following administration (76). Evidence suggests that B and T cells are not required for MPLA-mediated protection to *S. aureus*, neither were recruited monocytes; conversely, depletion of macrophages or neutrophils resulted in loss of MPLA-induced survival benefit (63). MPLA treatment enhances leukocyte recruitment, bacterial clearance, antimicrobial functions, and attenuates inflammation which all likely play a role in resistance to infection (65, 67). It is important to highlight that MPLA similarly enhances human neutrophil responses characterized by increased chemotaxis and bacterial killing (108). Additionally, MPLA stimulates the adaptive immune response whereby it increases antibody titers up to 20-fold (109) and thus is used as an adjuvant in malaria (AS01), human papillomavirus (HPV), and hepatitis B (AS04) vaccines (110, 111).

With their immunostimulatory properties, it is unsurprising that several synthetic TLR4 agonists have been developed with the goal of clinical translation. One promising class of synthetic TLR4 agonists are phosphorylated hexa-acyl disaccharides (PHADs) which are similar in structure to MPLA as they have only one phosphate group (16). PHADs similarly bind TLR4 and activate both MyD88- and TRIF-dependent signaling. Hernandez and colleagues recently showed that treating mice with PHADs confers protection to *P. aeruginosa* and *S. aureus*, both of which are of high clinical relevance (77). They found the survival benefit to be associated with increased bacterial clearance, an effect which was observed up to 10 days after treatment. Further, treatment with PHADs increased leukocyte recruitment and antimicrobial functions while attenuating systemic and local levels of proinflammatory cytokines (77).

Another synthetic lipid A TLR4 agonist, aminoalkyl glucosamine 4-phosphate (AGP), was first found to possess immunostimulatory properties two decades ago (112). Initially studied for their potential as a vaccine adjuvant (80), later studies demonstrated that AGPs confer protection to an otherwise lethal influenza challenge as well as to *Listeria monocytogenes* infection which were associated with increased bacterial clearance (78). Intranasal administration of AGP either before or after infection with the Gram-negative pathogen *Francisella tularensis* resulted in increased survival, and interestingly the survivors were protected against rechallenge with aerosolized *Francisella novicida* (113). Likewise, intranasal administration of AGPs prior to challenge with *Yersinia pestis* also extended time to death which was correlated with cytokine production and decreased bacterial load in the lung (80). Evidence suggests that TLR4 signaling *via* activation by AGPs induces NF-κB and IRF-3 signaling independent of the co-receptor CD14 (114).

Additionally, outer membrane vesicles (OMVs) with low endotoxicity by modification of lipid A of LPS (fmOMV) may increase protective benefit of intranasally administered influenza vaccine (115). Further, fmOMV confers protection against a lethal dose of pandemic viruses (H1N1, PR8, H5N2, and highly pathogenic H5N1) which is dependent on macrophages but independent of neutrophils. Treatment with fmOMV increased macrophage recruitment and production of type I IFNs without observance of adverse effects (81).

Beyond lipid A compounds, other natural and synthetic TLR4 ligands have been investigated. Fimbriae H protein (FimH) is the receptor-recognizing element of the adhesive organelle type 1 fimbriae on uropathogenic *E. coli* (UPEC) (116). Mice which receive FimH intranasally are resistant to influenza infection through increased recruitment of neutrophils and production of proinflammatory cytokines (TNF-α and IL-12) and chemokines (RANTES) in a macrophage-independent manner (82). Fusion of fimH with the MR/P fimbriae protein MrpH from *P. mirabilis* (MrpH.FimH fusion protein) conferred higher protection to UPEC and *P. mirabilis*, associated with reduced bacterial burden in the bladder and kidney as well as increased neutrophil recruitment (83).

Two classes of synthetic small molecule agonists have been studied with modest therapeutic potential. Neoseptin-3 is a more potent TLR4 agonist than LPS despite being structurally unique (117, 118). Neoseptin-3-activation of TLR4 signaling results in MyD88- and TRIF-dependent signaling and downstream activation of NF-κB and IFN-β but not IFN-α (118). Evidence suggests that Neoseptin-3 may hold strong vaccine adjuvant properties whereby mice immunized by ovalbumin (OVA) together with the compound had increased OVA-specific IgG production 21 days later when compared to immunized vehicle controls mice. However, these compounds failed to induce TLR4 signaling in human THP-1 monocytes, thus calling into question their therapeutic potential (118).

#### Toll-Like Receptor 5

TLR5 recognizes bacterial flagellin (119) and signals through MyD88, culminating in the production of inflammatory mediators. Studies have shown that bacterial flagellin is indeed an immunomodulatory agent. Mucosal administration of flagellin conferred resistance to *S. pneumoniae* lung infection whereby flagellin treatment increased bacterial clearance which was associated with increased neutrophil mobilization independent of B- and T-cells (120). More recently, this research group showed that sublingual administration of flagellin also effectively protects against pneumonia (85). Demonstrating cross-protection, treatment with bacterial flagellin was able to prevent infection with or cure ongoing infection of rotavirus in mice independent of adaptive immunity (84).

Bacterial flagellin also restores antibiotic-impaired innate immunity (121) and improves efficacy of antibiotics in the treatment of influenza virus or pneumonia (122). Thus, bacterial flagellin may be highly useful in prevention of infection in immunocompetent and immunocompromised individuals, as well as a strategy to combat antibiotic resistant microbes and boost efficacy of current antibiotic drugs.

### Immunomodulation *via* Targeting Intracellular Toll-Like Receptors

Numerous TLR agonists which activate intracellular TLRs have also been widely studied for their potential application as immunomodulators that trigger trained immunity, which are summarized in **Table 2**.

**Table 2:** Agonists which trigger trained immunity *via* intracellular TLRs.

TLR	Agonist	Route of Administration	Infectious Model	Antimicrobial Response	Reference
TLR3	Poly I:C & derivatives	*i.p.*	Punta Toro virus (*s.c.*)	↓ Organ injury (liver)	Gowen et al. (123)
		*i.p.*	HSV-1 (*i.n.*)		Boivin et al. (124)
		*i.n.*	Mouse-adapted SARS-CoV (*i.n.*)	↓ Organ injury (lungs)	Kumaki et al. (125)
				↓ Viral load (lungs)	
		*i.p.*	*E. coli* (*intracranial*)	↓ Viral load (systemic, cerebellum, and spleen)	Ribes et al. (126)
				↑ NK cell recruitment*	
				↑ INF-γ (brain and spleen)*	
				*In neutropenic but not immunocompetent hosts	
	CRL1505	Oral	Respiratory syncytial virus (RSV; *i.n.*)	↓ Viral load	Chiba et al. (127)
				Attenuation of Th2 reactions	
		*i.n.*	Respiratory syncytial virus (RSV; *i.n.*)	↓ Viral load	Tomosada et al. (128)
				↓ Viral replication	
				↓ Organ injury (lungs)	
		*i.n.*	Influenza A (*i.n.*)	↓ Viral load	Zelaya et al. (129)
				↓ Organ injury (lungs)	
				↑ Lymphocytes and DCs (lungs)	
		*i.n.*	Primary RSV (*i.n.*) + secondary *S. pneumoniae* (*i.n.*)	↑ Alveolar macrophages and T cells (lungs)	Clua et al. (130)
TLR7	1V270 (TMX201)	*i.n.*	*B. anthracis*, Venezuelan equine encephalitis virus, H1N1 virus (*i.n.*)	↑ Local, but not systemic, inflammation	Wu et al. (131)
	Imiquimod	*i.p.*	Polymicrobial sepsis (fecal-induced peritonitis)	↑ Neutrrophil recruitment	Wynn et al. (66)
				↑ Antimicrobial responses (phagocytosis)	
		*i.n.*	Influenza A (*i.n.*)	↓ Viral replication	To et al. (132)
				↓ Local inflammation	
				↓ Organ injury (lungs)	
	T7-EA	*i.p.*	Hepatitis B (*i.v.*)	↑ HBsAg-specific IgG2a titer & T-cell response	Hu et al. (133)
	CL097	*i.p.*	Hepatitis B transgenic mice	↑ HBsAg-specific T-cells (spleen)	Wang et al. (134)
	GS-9620	Oral	Hepatitis B chronically infected patients	↑ T-cell and NK cell responses	Boni et al. (135)
TLR9	CpG	*i.p.*	*L. major* (*oral*)	Shifts Th2 towards Th1 response	Zimmerman et al. (136)
		Intradermal	*L. amazonensis (intradermal)*	↓ Lesion size	Verthelyi et al. (137)
				↓ Parasite load	
		Mucosal (genital tract)	HSV-2 (intravaginal)	↓ Viral load (vaginal fluids)	Harandi et al. (138)
				T-cell dependent	
		*i.t.*	*K. pneumoniae* (*i.t.*)	↑ Bacterial clearance (systemic & lungs)	Deng et al. (139)
				↑ Neutrophils and lymphocyte recruitment	
		*i.p.*	*L. monocytogenes *(*i.p.*)	↑ CD4 & CD8 T cells	Ito et al. (140)
		*i.n.*	New World arenavirus Tacaribe (neurotropic virus: *i.n.*, *i.p.*, or *intracranial*)	↑ Ag-specific antibodies (IgG & IgM)	Pedras-Vasconelos et al. (141)
		*i.p.*	MRSA (*i.v.*)	↑ Bacterial clearance	Kim et al. (142)
				↓ Organ injury (lung, kidney, spleen)	
				↑ Lymphocyte recruitment	
				↑ Bacterial-reactive antibodies	

i.n., intranasal administration; i.p., intraperitoneal injection; i.t., intrathecal administration; i.v., intravenous injection; s.c. subcutaneous injection.

#### Toll-Like Receptor 3

TLR3 is localized to the intracellular compartment and recognizes viral dsRNA, including that produced during replication of ssRNA viruses or self-RNAs released from damaged cells (86, 143). Stimulation of TLR3 results in direct interaction with TRIF for downstream activation of IRF3 and modest activation of NF-κB (144). TLR3/TRIF activation culminates in production of type I IFNs and inflammatory cytokines for killing of invading viruses and has also been found to be important in cross-priming CD8+ T cell responses in a virus-specific manner (21, 145). As TLR3 signaling is MyD88-independent, the safety and immunostimulatory properties of TLR3-specific agonists is unique among other TLR immunomodulators (146).

The TLR3-activating synthetic dsRNA molecule poly-inosinic:poly-cytidylic acid (poly I:C) was first discovered to confer protection against subsequent viral challenge in 1969 (147). More recently, it was found that intraperitoneal administration of poly I:C 3 days prior to challenge with *E. coli* K1 meningitis in neutropenic mice resulted in increased recruitment of NK cells, production of RANTES and IFN-γ, and decreased bacterial burden (126). Pre-treatment with poly I:C resulted in survival benefit of neutropenic but not immunocompetent mice. In another model of infection, poly I:C conferred anti-viral properties (148) which lead to neuroprotection in a mouse model of HSV-1 encephalitis (124). In a unique oyster model, poly I:C injection protected the organism against subsequent environmental infection by mitigating viral replication which persisted for at least 5 months (148). It is suggested that the length of the dsRNA dictates distinct antimicrobial functions dependent on cell type which should be considered during study design and data interpretation (149). It has been posed that the difference in TLR3-mediated protection against viral infection compared to bacterial infection may be due to the production of type I IFNs which impairs bacterial clearance (15). Indeed, intranasal administration of poly I:C prior to challenge with *S. pneumoniae* and methicillin-resistant *S. aureus* increased susceptibility to infection (150).

Since its creation 5 decades ago, analogs of poly I:C have been rapidly developed in effort to reduce toxicity (146). The substitution of a uridylic acid at a molar ratio of 12:1 in the synthesis of the poly C strand results in poly I:C_12_U which is more rapidly metabolized *in vivo* (151). Pre-treatment with poly I:C_12_U protects against subsequent viral challenge more effectively than poly I:C (123). Interestingly, a protective benefit has also been observed when the molecule was administered 2 days after viral myocarditis infection (152). In a clinical trial in which poly I:C_12_U was administered to HIV-infected patients, immune function was restored or stabilized (153); however, clinical investigation did not progress past phase II clinical trials (154). Poly I:C_12_U also holds promise as a vaccine adjuvant as it increases efficacy of intranasal H5N1 immunization (155) and intradermal HSV-2 immunization, which conferred resistance to subsequent otherwise lethal HSV-2 infectious challenge (156). Demonstrating its safety, this compound has been developed as a therapy for chronic fatigue syndrome (157, 158) that is approved in Argentina and has been approved for early access program in the European Union and Turkey, although it does not currently have FDA approval in the United States (Rintatolimod, tradename Ampligen).

Two other chemically stabilized analogs of poly I:C have demonstrated promising immunostimulatory properties. The first being poly IC : LC (termed Hiltonol) which has been shown to protect rhesus monkeys from several viruses including yellow fever, Rift Valley fever, and rabies (159, 160) and has conferred protection against highly viral strains of H4N1 and influenza in mice (161, 162). Importantly, it was found in 2017 that intranasal administration of poly IC : LC 24 h prior to or 8 h after an otherwise lethal challenge with SARS-CoV conferred survival benefit as well as reduced lung hemorrhage scores and lung viral titers in mice (125). Additionally, this molecule has been investigated for its potential in boosting immunity of HIV-infected patients which induced transient innate immune responses, suggesting application as a vaccine adjuvant may be appropriate (163). This line of investigation is being pursued whereby administration of poly IC : LC alongside an antigen stimulates a ‘live virus vaccine equivalent’ effect whereby antigen-presenting cells (APCs) are activated, T lymphocyte response is elicited, memory T and B cells are generated, and T_eff_/T_reg_ ratios are increased (164). Keyhole limpet hemocyanin (KHL) or HPV vaccines elicited significantly elevated antibody responses and Th1 immune responses when administered with poly IC : LC in rhesus Macaques (165).

The second poly I:C stabilized analog is PIKA which has been shown to protect mice against an array of influenza viruses along with decreased viral burden in the lungs and increased recruitment of macrophages, neutrophils, and plasmacytoid DCs (166). However, application of PIKA has mostly focused on its potential as a vaccine adjuvant for H5N1 (167–169), Hepatitis B (HBsAg) (170), and rabies (171), the latter of which underwent phase II clinical trials with moderate success (172).

Beyond the poly I:C class of TLR3-stimulating molecules, oral administration of purified *L. rhamnosus* CRL1505 peptidoglycan confers resistance to RSV infection associated with decreased viral loads in the lungs and augmented cytokine responses (127). Protection against RSV and subsequent secondary infection to pneumococcal pneumonia was found to be dependent on TLR3 (128, 130) and macrophages (173). In an immunocompromised-malnourished model, it was found that immunostimulatory properties of CRL1505 peptidoglycan extended beyond augmentation of innate immunity. Administration of CRL1505 enhanced the Th2 response and recovery of B cells after *S. pneumoniae* infection (174). The investigators also found that intranasal administration of CRL1505 prior to challenge with influenza virus was associated with reduced pulmonary injury and viral loads in the lungs *via* regulation of pro-inflammatory cytokines and increased levels of type I IFNs (129).

Together, TLR3 agonists hold strong promise, especially in protection against viral infections and for vaccine adjuvant strategies. However, careful attention needs to be paid as to the potential propagation of bacterial infections by TLR3-induced production of type I IFNs.

#### Toll-Like Receptor 7

The endosomally located TLR7 recognizes ssRNA and often plays a role in responding to viral infections through MyD88-dependent signaling. The small molecule 1V270 (also designated TMX201) is a TLR7 ligand conjugated with a phospholipid that has been shown to protect mice from an otherwise lethal infection with *Bacillus anthracis*, Venezuelan equine encephalitis virus, and H1N1 influenza virus (131). 1V270-mediated protection was associated with increased cytokines and chemokines in bronchial alveolar lavage fluids but not in circulation.

The Imidazoquinoline compound Imiquimod is a low molecular weight compound which selectively activates TLR7. Imiquimod is an FDA approved immune response modifier for the topical treatment of genital warts caused by HPV (175). Imiquimod has also been found to be protective against influenza A infection in mice which was associated with reduced viral replication, airway inflammation, proinflammatory cytokine production, and preservation of body weight (132). Neonates were also protected against polymicrobial sepsis when infection was initiated 24 h after treatment (66). It is also important to note that imiquimod may be an effective vaccine adjuvant strategy for influenza (176).

Additionally, several TLR7 agonists have been shown to improve immunity of hepatitis B-infected hosts and are implicated as potential HBV vaccine adjuvants. When administered together with an alum adjuvant and recombinant hepatitis B surface antigen (HBsAg) protein, the novel TLR7 agonist T7-EA induced HBsAg-specific antibody and restored T-cell responses in a murine model (133). Similar improvement of HBsAg-specific T-cell function was observed by immunizing HBV-transgenic mice with a TLR7/TLR8 agonist (CL097)-conjugated HBV protein (134). In a prospective clinical study, it was found that oral administration of the TLR agonist GS-9620 increased T-cell responses to HBV peptides demonstrated by increased cytokine production; however, it failed to reduce serum HBsAg levels (135).

#### Toll-Like Receptor 9

TLR9 is expressed in DCs, monocytes, macrophages, and B cells and recognizes bacterial and viral DNA. Upon ligand binding, it signals through MyD88 directly; however, it is important to note that signaling depends on intracellular localization as a mechanism to fine-tune the immune response. In resting cells, TLR9 is localized to the ER (177). Recently described in detail by Marongiu et al., trafficking of TLR9 is controlled by the multimembrane protein unc-93 homolog B1 (UNCB1) which is required for the receptor to leave the ER and traffic to the Golgi (178). After delivery to the plasma membrane, the adaptor protein AP-2 is recruited to mediate internalization of the receptor in a clathrin-dependent mechanism (179). In parallel to internalization of TLR9, the ligand must also be endocytosed. TLRs are then localized to early endosomal compartments and the pathway bifurcates to either IRF7 signaling endosomes or NF-κB signaling endosomes (180), which are determined by AP-3 (181). Thus, the localization of TLR9, presence of co-receptors and co-factors, as well as trafficking of the ligand itself all influence downstream signaling and determine whether pro-inflammatory cytokines or type I IFNs are produced. Such intricate control also prevents recognition of self-DNA to prevent autoimmune dysfunction.

CpG oligodeoxynucleotides (CpG ODNs) are synthetic molecules which mimic bacterial DNA and stimulate TLR9. Pre-treatment with CpG has been found to protect mice from *Leishmania major* infection by shifting immune responses from Th2 towards Th1 (136). CpG conferred survival benefit to *Leishmania major* and *F. tularensis* for up to 2 weeks independent of the route of infection (182). Intrathecal administration of CpG 48 h prior to *Klebsiella pneumoniae* infection resulted in increased survival, associated with reduced bacterial burden in the lungs and circulation, increased recruitment of neutrophils, NK cells, γδ-T cells, and augmented inflammatory response (139). Interestingly, treatment of methicillin-resistant *S. aureus* (MRSA)-infected mice with CpG improved survival (142). Beyond protection against bacterial microbes, CpG conferred survival benefit to viral challenge by HSV-2 which was associated with decreased viral replication (138) *via* augmentation of the innate immunity (183). CpG has also conferred protection of neonate mice challenged with *Listeria* infection (140) as well as neurotropic Tacaribe Arenavirus which was associated with decreased viral load, increased antigen-specific antibodies, and NO production *via* NO synthase expression (141). CpG-mediated protection was preserved in T cell-depleted immunocompromised mice (182). HIV-infected macaques treated with CpG prior to challenge with *Leishmania* exhibited decreased lesion size and parasite load (137). The two latter studies clearly demonstrate the clinical potential of CpG to protect immunocompromised populations against opportunistic infections.

In 2017, the FDA approved the use of CpG 1018 as a vaccine adjuvant in a hepatitis B vaccine (Heplisav-B) which has increased efficacy of the vaccine, thus reducing the prior three-dose strategy to a two-dose strategy (184). CpG 1018 increases antibody concentrations, stimulates helper (CD4+) and cytotoxic (CD8+) T cells, boosts T and B cell memory responses, and shifts T cells towards a Th1 response. Researchers have found that the 2-dose HBV vaccine strategy with CpG as the adjuvant compared to the 3-dose strategy with aluminum hydroxide was more effective in patients aged 60–70 years old with type 2 diabetes mellitus (185), a population which typically demonstrates reduced immunogenicity compared to younger and/or non-diabetic populations. Thus, CpG may be a beneficial agent to boost immune responses in vulnerable patient populations.

### Other Pathogen Associated Molecular Patterns Which Trigger Trained Immunity in a Toll-Like Receptor-Associated Mechanism

#### Bacillus Calmette-Guerin Vaccine

The Bacillus Calmette–Guerin (BCG) tuberculosis vaccine is the most used vaccine globally which has been demonstrated to confer T cell-independent cross-protection against fungal infection with *C. ablicans* or with the parasite *Schistosomiasis mansoni* (186, 187). After adjusting for age and other vaccines, the BCG vaccine is associated with a significantly lower mortality ratio among infants in Ginea-Bissau (188). In animal studies, BCG-mediated non-specific protection lasts for at least 3 months and is independent of T and B cells (189) and there is evidence that protection may last up to a year (190). Although the immunization effect of the BCG vaccine against *M. tuberculosis* requires adaptive immunity, namely T cell activation, the initial response to BCG is through the innate immunity whereby TLR2 and TLR4 and downstream MyD88-dependent signaling are activated (191). The resulting activation of the NF-κB pathway serves as the link between the innate and adaptive response. Therefore, the BCG vaccine does activate the innate immune response and may be responsible for driving broad protection.

#### β-Glucan

Fungal β-glucans are a promising class of molecules which trigger trained immunity, although the immunostimulatory properties differ depending on the strain from which they were isolated (192). These naturally derived molecules have been found to confer protection against a model of *E. coli* peritonitis (193), *S. aureus* (194), influenza (195), and MRSA (196) and were associated with increased leukocyte recruitment and antimicrobial functions. Treatment of burn-injured mice with glucan phosphate prior to wound infection with *P. aeruginosa* improved survival, attenuated cytokine production, and decreased bacterial load at the burn wound (197).

β-glucans bind their specific PRR dectin-1 which results in downstream inflammasome activation. However, β-glucan-mediated production of inflammatory cytokines and reactive oxygen species (ROS) is dependent on the cooperation between dectin-1 and TLR2 (198). Further, TNF-α production in response to zymosan or live fungi is dependent on MyD88 (199). Thus, β-glucan-mediated trained immunity is dependent on the synergism of dectin-1 and TLR2.

#### CL429

One research group is investigating the potential immunostimulant properties of CL429, which is a novel chimeric compound that was designed to stimulate both TLR2 and NOD2 by covalently linking the NOD2 ligand Murabutide with the TLR2 ligand Pam_2_C (200). Initially studied as a vaccine adjuvant, they went on to find that CL429 confers protection against pneumovirus (PVM) infection associated with attenuated inflammation (201) and against leptospiral infection for up to 3 months *via* increased proinflammatory cytokine and chemokine production (202).

#### CpG-Oligodeoxynucleotide : AG-OVA Nanoparticles

Similar in approach as the CL429 molecule, the TLR9-activating ligand CpG ODN was crosslinked with the dectin-1/TLR2 stimulating agonist β-glucan-Ovalbumin resulting in CpG-OND : AG-OVA dual-targeting nanoparticles as a vaccine adjuvant strategy (203). Investigators found that the nanoparticles enhanced APC maturation and induced robust Th1 and Th2 responses similar to that triggered by Freund’s adjuvant but without the toxicity. Although this novel compound demonstrates promise as a vaccine adjuvant, it would also be of interest to investigate whether it confers broad protection to infection which may be more profound than that mediated by CpG or β-glucan alone, both of which are strong immunomodulators.

### Cross-Protection Between Infections

As the BCG vaccine seemingly mediates cross-protection against pathogens besides tuberculosis, evidence suggests that some infections also confer cross-protection which is, at least in part, due to trained immunity (10). For example, administration of an attenuated strain of *C. albicans* conferred host resistance to subsequent challenge with the Gram-positive bacteria *S. aureus*; this phenomenon was found to be independent of T cells but was dependent on macrophages (204). Interestingly, several observations have suggested that viral infections may trigger a similar cross-protection benefit. Barton et al. demonstrated that latent herpesvirus was associated with protection against bacterial *L. monocytogenes* and against bacterial *L. monocytogenes* and *Y. pestis* which was similarly dependent on macrophages (205). This observation was confirmed by others who elucidated that herpesvirus-induced protection against bacterial infection is transient (approximately 5 months) despite stable viral load (206). NK cells are also key mediators of cross-protection whereby they expand during the initial infection and are primed to undergo a second expansion as well as produce more cytokines upon a secondary infection (207). More recently, a Singapore study of military recruits within a 5-year period showed that men infected with influenza were protected against subsequent infection with adenovirus (79). It should be noted, however, that cross-protection between infections is generally considered to be dependent on both non-specific reprogramming of innate immunity as well as activation of memory T cells.

### Toll-Like Receptor Antagonists as Potential Immunomodulatory Strategies for Treatment of Chronic Infectious Diseases

It is clear that TLR agonists hold strong therapeutic potential to mediate host resistance to subsequent infection; however, this is only one of many potential therapeutic applications of TLR immunomodulators. As TLR signaling cascades culminate in robust inflammation, TLR antagonists are under development for the treatment of chronic infectious and inflammatory diseases (208). To date, these compounds are generally designed to bind the TLR, thus preventing the binding of agonists responsible for driving inflammation (209–211). The TLR4 antagonist Eritoran (E5564) reached Phase III clinical trials for the treatment of sepsis. This synthetic lipid A analogue which prevents LPS from activating TLR4, hypothetically preventing propagation of systemic inflammatory response syndrome (SIRS) characteristic of sepsis. Preclinical and early clinical studies with the compound showed promising anti-inflammatory results in response to LPS (212–214), however the study failed to meet its target end-point in phase III (215). Similarly, another TLR4 antagonist designated Resatorvid (TAK-242) was also studied in the treatment of sepsis and reached Phase III of the clinical trials, however it failed to attenuate inflammation in septic patients (216).

Recently, the antimalarial drugs chloroquine and hydroxychloroquine have been under investigation for treatment of COVID-19 with the hypothesis that these drugs will prevent glycosylation of the angiotensin-converting-enzyme 2 (ACE2) as well as inhibit endosomal TLR activation (217, 218). Initial *in vitro* studies showed potent antiviral activity (219). Randomized trials have not shown improved clinical outcomes in the hydroxychloroquine-treated COVID patients (218, 220, 221). In another study, the Bruton tyrosine kinase (BTK) inhibitor acalabrutinib was administered to COVID patients for 10–14 days which seemed to improve patient outcomes as indicated by oxygenation (222).

Although clinical trials using TLR antagonists in the treatment of severe infection have been unsuccessful to date, critical information has been gained from these investigations which provide a strong foundation for future studies. Importantly, this class of compounds is also being widely studied for treatment of chronic inflammatory conditions such as rheumatoid arthritis and autoimmune disorders. One key finding of the clinical trials studying the TLR4 antagonists Eritoran and Resatorvid was that the compounds were well-tolerated (215). Continued drug discovery efforts *via* high throughput screening and alternative approaches such as targeting the transcriptional regulation of TLRs to suppress their expression rather than direct inhibition of the receptor may move the field forward (223). These efforts will be also be supported by continued elucidation of TLR signaling mechanisms and immune responses.

## Metabolic and Epigenetic Reprogramming as the Basis for Toll-Like Receptor Agonist-Induced Trained ImmunitY

Upon inflammatory stimulation, innate leukocytes undergo metabolic reprogramming that is characterized by augmentation of glycolysis and mitochondrial oxidative phosphorylation to meet the increased energy demands for combating an infection (14, 224). Previous investigations aimed at deciphering the molecular mechanisms of trained immunity-mediated protection against infections have predominantly used the fungal ligand β-glucan (225, 226). Reviewed in detail by Netea and colleagues, metabolic reprogramming and epigenetic modifications are the key mechanisms of β-glucan-induced trained immunity (226). It has been shown that Akt/mTOR/HIF-1α signaling is critical in β-glucan induced augmentation of glycolysis in monocytes (225). As opposed to the breadth of mechanistic understanding of trained immunity induced by β-glucan, the molecular mechanisms underlying TLR ligand-induced training of leukocytes are an evolving field. Our studies show that treatment with the TLR4 ligand MPLA not only increases glycolysis but also augments mitochondrial oxidative phosphorylation and mitochondrial biogenesis in concert with increased antimicrobial functions of macrophages (63). Further, stimulation of macrophages with the classic TLR4 ligand LPS reprograms mitochondrial metabolism leading to increased accumulation of tricarboxylic citric acid (TCA) cycle metabolites that play an important role in TLR agonist-mediated trained immunity (227, 228).

The finding that a variety of TLR ligands have the ability to mediate protection against infection from a broad array of organisms which activate distinct TLRs, including Gram-positive and Gram-negative bacteria, fungi, and viruses, demonstrates that TLR activation has the ability to offer cross-protection against diverse pathogens. Both MyD88 and TRIF activation have been implicated in facilitating signaling-driven metabolic and epigenetic alterations induced by TLR ligands (229), but little is understood about the roles of these signaling pathways in initiating trained immunity. This raises the question as to whether activation of trained immunity by TLR ligands is mediated through common signaling pathways. Future studies providing insight into these common pathways would pave the way for the discovery of a multitude of potential TLR ligand-based therapeutics to improve resistance to infection. The following sections will provide a succinct overview of leukocyte metabolic reprogramming and epigenetic modifications as the basis for TLR ligand induced trained immunity (**Figure 2**).

### Toll-Like Receptor Ligand-Induced Metabolic Reprogramming of Innate Leukocytes

Stimulation of macrophages with LPS increases glucose uptake and glycolytic capacity mediated *via* stabilization and upregulation of hypoxia-inducible factor 1- α (HIF-1α) (230–232). Increased glycolytic capacity serves to rapidly generate ATP and provide essential precursors for synthesis of amino acids, lipids, and nucleotides that are necessary for optimal effector activities and cell viability under stress conditions (233). Our studies have shown that deletion of HIF-1α or inhibition of mammalian target of rapamycin (mTOR; known to stabilize HIF-1α) attenuates MPLA-induced increased glycolysis, abolishing the protective effect of MPLA against infection (63, 76).

Along with augmented glycolysis, reprogramming of mitochondrial metabolism plays a key role in modulating the inflammatory response of innate leukocytes. LPS-induced activation of macrophages introduces ‘breaks’ in the TCA cycle at the levels of isocitrate dehydrogenase and succinate dehydrogenase (SDH), leading to increased accumulation of citrate and succinate (234). Studies from our laboratory also show that MPLA treatment cause an early reduction in TCA cycle flux between citrate and α-ketoglutarate leading to increased accumulation of citrate (63). Citrate is diverted towards generation of itaconate *via* increased immunoresponsive gene 1 (Irg1) enzyme expression (235). Itaconate is being widely investigated for its direct antimicrobial and anti-inflammatory effects. The direct antimicrobial effect of itaconate is mediated *via* inhibition of the microbial enzyme isocitrate lyase (236). Itaconate has been shown to inhibit the growth of numerous pathogens including *M. tuberculosis, S. aureus*, *Legionella pneumoniae*, *Acinetobacter baumanii*, and *Salmonella enterica* (235, 237, 238). Itaconate can be transported into the phagosome, where it limits microbial growth (239), implying a role for phagolysosomes as a critical site for itaconate’s antimicrobial effects. The synthetic itaconate analog, 4-octyl-itaconate (4OI) exerts anti-inflammatory effects and potently activates the NF-E2-related factor 2 (Nrf2) pathway (240) which regulates the expression of cytoprotective proteins and plays a critical role in redox homeostasis (241). A study by Swain et al. shows that endogenous itaconic acid fails to activate Nrf2 as compared to 4OI, and that synthetic itaconate analogs do not recapitulate the effects of endogenous itaconic acid (242). However, endogenous itaconic acid is anti-inflammatory and reduces IL-1β production similar to 4OI (240, 242). In contrast to these findings, treatment with β-glucan does not induce significant levels of Irg1 and itaconate in human monocytes, and pretreatment with 4OI diminishes β-glucan induced trained phenotype (243). However, it is important to note that β-glucan focused studies relied on measuring cytokine responses alone as a key for demonstrating the lack of effect of itaconate on β-glucan-induced trained immunity although cytokine responses to an inflammatory stimuli may not correlate with actual protective response (76).

Beyond the role of citrate in serving as a precursor for itaconate, our studies using MPLA-stimulated macrophages have shown that citrate transported into the cytosol replenishes mitochondrial oxaloacetate pools and fuels a sustained increase in mitochondrial TCA cycle flux (63). Upon TLR4 stimulation of macrophages, inhibition of SDH activity by itaconate and increased TCA cycle flux also results in succinate accumulation (231, 244). Increased succinate levels and inhibition of SDH activity stabilize HIF-1α and increase mitochondrial ROS generation which lead to an enhanced inflammatory response (231, 244). Mitochondrial ROS aid in microbial clearance (245); however, this phenomenon requires further investigation. Importantly, MPLA-induced increase in TCA cycle flux is associated with enhanced macrophage antimicrobial effects and protection against infections (63). Therefore, metabolic reprogramming plays a critical role in TLR ligand induced trained immunity-mediated protection against infections.

### Role of Epigenetic Modifications in Toll-Like Receptor Ligand-Induced Trained Immunity

Exposure to inflammatory stimulus or pathogens also causes epigenetic reprogramming in innate leukocytes reflected by alterations in the histone acetylation and methylation status (246). The major histone modifications including acetylation (H3K27ac) and methylation (H3K4me3) in monocytes exposed to β-glucan persist even 7 days after removal of the initial stimulus and are strongly associated with metabolic reprogramming (225). A study by Saeed et al. showed that LPS and β-glucan induce diverse and opposing alterations in the epigenome with β-glucan showing a greater degree of *de novo* H3K27ac modifications in gene loci encoding for inflammatory responses (247). Alternatively, LPS stimulation of human monocytes acutely induces a strong acetylation of H3K27 around Irg1 gene locus within 1 h of stimulation which is associated with increased expression of Irg1 (243).

Metabolic reprogramming and epigenetic modifications are tightly interconnected. Fumarate accumulates in monocytes stimulated with β-glucan *via* glutamine anaplerosis. Increased fumarate levels have been shown to downregulate the activity of histone demethylase KDM5 (248). In turn, decreased KDM5 activity upregulates trimethylation of H3K4 at promoters of genes encoding pro-inflammatory cytokines (248). α-ketoglutarate stimulates the jumonji domain containing family of the lysine demethylase enzyme JMJD33. Further, a high α-ketoglutarate/succinate ratio favors anti-inflammatory phenotype in macrophages (249).

The post-translational modification of succinylation arising from the addition of succinate to the protein lysine residues supports a pro-inflammatory state in macrophages (231). Succinylation is known to occur on histone lysine residues in human cells (250). However, the role of histone succinylation in the context of trained immunity is currently unknown and remains an important question to be addressed in future studies. A study by Zhang et al. showed that lactate can also bind to histone lysine residues (lactylation) and demonstrated 28 distinct histone lactylation sites (251). This study showed that LPS-induced increase in macrophage lactate levels *via* increased glycolysis sets in motion a lactylation epigenetic program which directs the expression of genes involved in alternative anti-inflammatory activation state in macrophages (251). The influence of accumulated metabolites during trained immunity is just beginning to be explored and needs further characterization.

The traditional school of thought is that treatment with TLR ligands such as LPS induces a state of immune tolerance, while leukocytes exposed of β-glucan produce a heightened response to secondary stimulation (trained phenotype) classically reflected by cytokine production (252). However, as discussed, treatment with clinically applicable TLR agonists protect against a broad array of infections, implying that TLR ligands also induce robust trained immunity. One potential explanation for this discrepancy in the literature regarding whether TLR ligands induce a state of tolerance or training may be due to the reliance on cytokine production upon exposure to a secondary stimuli which has more recently been shown to not be uniformly indicative of antimicrobial immunity (76). Future studies aimed at detailed characterization of TLR agonist-induced epigenetic reprogramming and defining its link with metabolic reprogramming will be critical in elucidating the mechanisms of TLR agonist-induced trained immunity.

## Toll-Like Receptor Agonist-Induced Trained Immunity: A Clinical Perspective

### Potential Adverse Consequences of Immunostimulation by Toll-Like Receptor Agonists

It is important to thoroughly consider that activation of the immune system may have deleterious consequences and requires careful study to identify the clinical situations in which TLR-mediated immunomodulation are most appropriate. Sepsis and septic shock yield a proinflammatory response that results in organ injury; however, survivors demonstrate an immunosuppressive phenotype that results in secondary infections and increased mortality (253, 254). Likewise, a potentially harmful outcome of TLR agonist treatment is tolerance to subsequent exposure of endotoxin, particularly in the setting of prolonged LPS exposure or treatment, a phenomena also termed as immunoparalysis (255, 256). Aberrant activation of TLR signaling by PAMPs or DAMPs, mutations of TLR signaling molecules, or failure of self-recognition mechanisms are responsible for development of several diseases such as autoimmune, chronic inflammatory, and allergic diseases (257). In the field of oncology research, adverse effects from TLR immunotherapy have been linked to unintended expansion of adaptive leukocytes, such in B-cell lymphoma, where activation of TLR4 MyD88-dependent signaling may exacerbate the disease (258–260). Other adverse effects of treatment with TLR agonists have been described in cardiovascular medicine research where treatment with oxidized low-density lipoprotein and the BCG vaccine yield a dose-dependent response of proinflammatory cytokines. These mediators damage human coronary smooth muscle cells and increase atherosclerosis which was found to be TLR2- and TLR4-dependent (261).

### Therapeutic Potential of Toll-Like Receptor Ligands Beyond Infection Resistance

Although there are some potential adverse effects of TLR immunotherapy which require consideration, there are several patient populations which may benefit from novel TLR agonist strategies. The oncology patient population presents a challenge due to immunosuppression. Most cancer-associated antigens are self-antigens and require immunostimulant adjuvants in addition to cancer-targeting strategies (262). In an H22 liver cancer murine model, administration of curdlan sulfate-matured tumor cell lysate-pulsed DCs was associated with an increase in CD80, MHC-1 and MHC-II expression, CD8+T cell infiltration, upregulated TNF-α and INF-γ transcription, and downregulation of TGF-β transcription in tumor tissues, and improved survival (263). In a separate study, the use of the TLR3 agonist Ampligen, a GMP-grade synthetic poly I:C derivative, was shown to mature human monocytes derived from DCs and sustained bioactive IL-12 production, and generate Th1 specific anti-cancer responses in peripheral blood T-cells obtained from cancer patients (262). In a study by Breckpot et al., the zinc finger protein A20 was downregulated in poly I:C treated DCs which led to sustained production of IL-6, IL-10, and IL-12p70, thus making poly I:C a candidate adjuvant for an anti-cancer immunotherapy (264). Likewise, intratumor administration of the TLR7 agonist 1V270 increased the ratio of M1 to M2 tumor-associated macrophages and was associated with improved survival (265). In addition to anti-cancer immunotherapy, TLR agonists have shown promise in reduction of ischemia reperfusion injury in cardiac myocytes (266, 267). Further, TLR therapy has also been studied in progressive diseases such as Alzheimer’s disease, where single or repeated treatment has been shown to reduce evidence of disease progression (268, 269).

Aside from innate immune cells, non-immune cells are capable of long-term memory, including hematopoietic, mesenchymal, and epithelial stem cells (270). There is an increasing body of evidence on how the microbiome influences immunity and how probiotic therapy modulates innate immunity (271). Interestingly, trained immunity may be a mechanism of the beneficial effects of probiotics. Probiotics have been found to augment innate immune function *via* receptor antagonism or expression, binding or expression of adaptor molecules, expression of regulatory signaling molecules, induction of micro-RNAs, and secretion of immunomodulatory proteins, lipids, and metabolites (271).

Trained immunity may even play a role in combating the ongoing COVID-19 pandemic. As one of the sequelae of COVID-19 infections include secondary respiratory infections, the BCG vaccine or β-glucan may be adjunct strategies to reduce morbidity and mortality by enhancing immunity (272, 273). Imiquimod, a TLR7 agonist, has also been proposed as a therapeutic adjunct for COVID-19 and related infections (274). In summary, there is a growing body of evidence focusing on trained immunity as a mechanism to enhance immunity against a broad array of infections which are common in critically ill patients, but also for several other patient populations as discussed above. Such application requires further investigation to elucidate the full potential of TLR agonist-based immunotherapies (**Table 3**).

**Table 3:** Clinical application of TLR immunomodulators.

Application of TLR Immunomodulator	Examples	Mechanism	Potential Therapeutic Outcome	Potential Adverse Consequences
Resistance to infection	TLR2 agonist Pam_2_Cys, TLR4 agonists MPLA & PHAD, TLR3 agonist poly I:C, TLR9 agonist CpG	Increased leukocyte recruitment and antimicrobial functions	Improved survival; reduced risk of nosocomial infections; reduced reliance on antibiotics	Chronic inflammation; autoimmune disease
Vaccine adjuvant	TLR4 agonist MPLA as an approved adjuvant in malaria (AS01), human papillomavirus (HPV), and hepatitis B (AS04) vaccines	Immune stimulation for increased antibody titers	Improved efficacy of vaccines and reduced dosing strategies	Discomfort at injection site; transient malaise
Cancer immunotherapy	TLR3 agonist poly I:C & derivatives; TLR7 agonist 1V270	T-cell activation and DC maturation	Antitumor immunity	Dose-limiting side effects (fatigue, malaise, fever)
Chronic infections & inflammatory diseases	TLR4 antagonist Eritoran to treat sepsis; TLR9 agonist Lefitolimod for reduction of HIV-1 viral reservoir	Antagonize TLR to prevent activation and downstream inflammation	Reduced inflammation and associated organ injury	Immune tolerance

### Clinical Trials Investigating Toll-Like Receptor Immunomodulators

Ongoing clinical trials investigating the adjuvant properties of TLR agonists constitute approximately double of those studying them as therapeutics (275), demonstrating that the immunomodulatory properties of these compounds are largely being harnessed for vaccine development. The application of trained immunity for vaccine development is highly attractive due to its potential to (1) increase nonspecific effector responses of innate immune cells, and (2) to activate DCs to enhance adaptive T cell responses to specific and nonrelated antigens (276). For example, a novel synthetic small molecule TLR7/8 agonist 3M-052 is now in a phase I clinical trial studying the safety and immunogenicity of the HIV-1 BG505 SOSIP.664 gp140 vaccine candidate (NCT04177355). Other TLR agonists currently in clinical trials as vaccine adjuvant strategies for HIV-1 include TLR3 agonist Poly ICLC (NCT02071095) and TLR9 agonists MGN1703 (NCT02443935) and CpG-7909 (NCT00562939). TLR7 compounds are under investigation as vaccine adjuvant strategies for hepatitis B as well, including Vesatolimod (GS-9620; NCT02166047) and R07020531 (NCT02956850). The TLR9 compound SD-101 is being studied as an adjuvant for chronic hepatitis C (NCT00823862).

On the other hand, the TLR9 agonist Lefitolimod in combination with neutralizing antibodies is in phase II trials studying its effectiveness in conferring reservoir reduction in HIV infection (NCT03837756) after phase I demonstrated its safety and effectiveness in improving both innate and adaptive immunity in HIV-1 infected patients (277). The TLR7 compound Imiquimod has completed phase II trials for its efficacy in treating human papillomavirus (HPV) when applied topically (NCT00941811).

In addition to the above listed clinical trials regarding investigation of TLR agonists as vaccine adjuvant strategies or drugs to fight infection, numerous TLR ligands are being investigated as immunomodulators to treat chronic inflammatory diseases, cancer, and autoimmune disorders, which can be found listed in Anwar et al. (275).

### Current Challenges to the Clinical Translation of Toll-Like Receptor Immunomodulators

Although there are several potential clinical applications of TLR immunomodulators as stand-alone therapies which are supported by a growing body of strong preclinical evidence, several knowledge gaps hinder progress of clinical translation. Much remains to be elucidated regarding duration of protection mediated by TLR agonists, and whether protection could be continued by repeated treatment once protection wanes. Further, the most effective but feasible route of administration is essential to identify, with oral and intranasal administrations likely most practical. Dosing and efficacy for various patient populations is also essential to understand, such as whether aged patients or those with comorbidities respond similarly to healthy young patients. In this regard, one limiting factor remains the use of healthy young animals for the vast majority of preclinical studies which does not recapitulate the clinical situation and therefore limits the amount of information that could be gained prior to starting clinical trials.

Another limiting factor is the striking lack of reporting of clinical trial data; therefore, scientists do not have all of the tools that otherwise could refine ongoing and future studies. Finally, as TLR agonists initiate inflammatory cytokine pathways, one glaring concern remains the potential of these compounds to trigger inflammatory or autoimmune disease. Thus, it is critical to continue elucidating TLR signaling mechanisms to identify potential therapeutic targets which may circumvent this concern in addition to conducting proper dosing studies aimed at avoiding induction of inflammation. Rapid scientific progress has been made since the discovery of the phenomenon of trained immunity and its potential therapeutic application. As the field drives forward to fill in these knowledge gaps, the goal of clinical translation of TLRs as immunomodulators holds strong promise to be realized.

## Concluding Remarks

Unfortunately highlighted during the ongoing SARS-CoV-2 pandemic, immunocompromised patients are highly susceptible to life-threatening infections. Beyond the current healthcare crisis, populations with insufficient immune responses fail to clear pathogens which results in opportunistic infections that are often difficult to combat due to the increasing prevalence of antibiotic resistance. With limited pharmacological tools, it is critical to develop new strategies aimed at combating infection. Here we have reviewed the potential application of TLR agonists as immunotherapies which trigger trained immunity and confer broad protection to microbes. Importantly, since immunomodulation targets the host response rather than the pathogen, development of microbial resistance is unlikely. TLR agonists have also shown promise as adjuvants for cancer-targeting immunotherapies. Moreover, our lab and others have demonstrated that such agonists may be highly useful as stand-alone therapies to protect against infection through boosting antimicrobial responses of innate leukocytes.

With their instrumental role in stimulating innate immunity and in activation of inflammatory responses, TLRs are tightly controlled by localization to the cell surface or endosomal compartment as well as complex downstream signaling pathways *via* MyD88- or TRIF-dependent cascades. Although much remains to be elucidated, TLR-mediated trained immunity seems driven by metabolic reprogramming and epigenetic modifications. It is critical to further elucidate the cell types, signaling pathways, and intracellular mechanisms responsible for conferring the beneficial protective effects of TLR agonists *via* trained immunity. Doing so will aid the translation of TLR-based immunotherapies to protect patients from potentially life-threatening infections.

## Author Contributions

The manuscript was conceptualized by AO and JB. AO wrote the sections on TLR signaling pathways, trained immunity triggered by TLR agonists, and drafted the figures. JF wrote the introduction and drafted the tables. NP wrote the sections on metabolic and epigenetic modifications, and AH contributed the section on clinical perspectives. JB supervised the drafting and performed the final editing. All authors contributed to the article and approved the submitted version.

## Funding

AO is supported by 5T32AI38932-02, NP is supported by 5T32GM108554-05, Shock Society Faculty Research Award, and Vanderbilt Faculty Research Scholar Award, AH by K08 GM123345, and JB by R01 GM121711.

## Conflict of Interest

The authors declare that the research was conducted in the absence of any commercial or financial relationships that could be construed as a potential conflict of interest.

## Glossary

ACE2: Angiotensin-converting-enzyme 2
AGP: Aminoalkyl glucosamine 4-phosphate
AP-1: Activator protein-1
APC: Antigen-presenting cell
BCAP: B-cell adaptor for PI3K
BCG vaccine: Bacillus Calmette-Guerin tuberculosis vaccine
BTK: Bruton’s tyrosine kinase
CDC: Centers for Disease Control and Prevention
CpG ODN: Cytosine-phosphate-guanine dinucleotides oligodeoxynucleotides
CLP: Cecal ligation and puncture
DAMP: Damage-associated molecular pattern
DCs: Dendritic cells
dsRNA: Double-stranded RNA
DUBA: Deubiquitinating Enzyme A
EGF: Epidermal growth factor
ER: Endoplasmic reticulum
FDA: U.S. Food and Drug Administration
FimH: Fimbriae H protein
fmOMV: Low endotoxicity outer membrane vesicles
H1N1: Influenza A subtype
H5N1: Influenza A subtype
avian flu: bird flu
H5N2: Influenza A subtype
avian flu: bird flu
HBV: Hepatitis B virus
HBsAg: Hepatitis B surface antigen
HCAI: Healthcare associated infections
HIV: Human immunodeficiency virus
HPV: Human papillomavirus
HSV: Herpes simplex virus
ICU: Intensive care unit
Ig: Immunoglobulin
IKK: IkB kinase
IKKi: IKK inducible gene
IRAK: Interleukin-1 receptor-associated kinases
IRF: Interferon regulatory factor
KHL: Keyhole limpet hemocyanin
LPS: Lipopolysaccharide
LRR: Leucine-rich repeat
MAL: MyD88-adaptor-like
MALP: Macrophage-activating lipopeptide
MAPK: Mitogen-activated protein kinase
MCP: Monocyte chemoattractant protein
miRNA: Micro RNA
MPLA: Monophosphoryl lipid A
mRNA: Messenger RNA
MrpH: MR/P fimbriae protein
MRSA: Methicillin-resistant *S. aureus*
MyD88: Myeloid differentiation 88
NADPH: Nicotinamide adenine dinucleotide phosphate
NEMO: NF-kB essential modifier
NF-κB: Nuclear factor-κb
NK cells: Natural killer cells
NO: Nitric oxide
NOD: Nucleotide oligomerization domain
OMV: Outer membrane vesicles
OVA: Ovalbumin
Pam2Cys: Peptide dipalmitoyl-S-glyceryl cysteine
Pam3Cys: Peptide tripalmitoyl-S-glyceryl cysteine
PAMP: Pathogen-associated molecular pattern
PHAD: Phosphorylated hexa-acyl disaccharides
PI3K: Phosphatidylinositol 3-kinase
Poly I:C: Polyinosinic:polycytidylic acid
PR8: Mouse-adapted H1N1
PRDX: Peroxiredoxin
PRR: Pattern recognition receptor
PVM: Pneumovirus
RAG: Recombination-activating gene
RANTES: Regulated upon activation, normal T cell expressed and secreted
RIP: Receptor-interacting protein
RSV: Respiratory syncytial virus
SDH: Succinate dehydrogenase
ssRNA: Single-stranded RNA
TAK: Transforming growth factor β–activated kinase 1
TANK: TRAF-associated NFκB activator
TBK: TANK-binding kinase
TCA cycle: Tricarboxylic citric acid cycle
T_eff_: Effector T cell
Th: T helper cell
THP: Tamm-Horsfall protein
TICAM: TIR domain-containing adaptor molecule
TIR: Toll/interleukin receptor
TIRAP: TIR domain-containing adaptor protein
TLR: Toll-like receptor
TMX201: Small molecule 1V270
TNF: Tumor necrosis factor
TRAF: TNF receptor-associated factor
TRAM: TRIF-related adaptor molecule
T_reg_: Regulatory T cell
TRIF: TIR-domain-containing adapter-inducing interferon-B
UNCB1: Unc-93 homolog B1
UPEC: Uropathogenic *E. coli*
